# Incidence trend analysis of tuberculosis in Khuzestan Province, southwest of Iran: 2010–2019

**DOI:** 10.1016/j.gloepi.2023.100118

**Published:** 2023-08-04

**Authors:** Seyed Mohammad Alavi, Mostafa Enayatrad, Bahman Cheraghian, Neda Amoori

**Affiliations:** a Infectious and Tropical Diseases Research Center, Health Research Institute, Ahvaz Jundishapur University of Medical Sciences, Ahvaz, Iran; b Clinical Research Development Unit, Bahar Hospital, Shahroud University of Medical Scinces, Shahroud, Iran; c Department of Biostatistics and Epidemiology, School of Public Health, Ahvaz Jundishapur University of Medical Sciences, Ahvaz, Iran; d Abadan University of Medical Sciences, Abadan, Iran

**Keywords:** Incidence, Tuberculosis, Epidemiology, Trend

## Abstract

**Objectives:**

Identifying the trend of diseases and its changes over time can be highly important in evaluating the extent and method of achieving strategies for controlling them, developing health indicators, and health planning. This study aimed to investigate the incidence of tuberculosis.

**Methods:**

As a repeated cross-sectional study in which the population under study was a census, this study involved all tuberculosis cases registered in 21 cities of Southwest of Iran, from 2010 to 2019. Data were obtained from the National System of Notification of Tuberculosis and included variables related to age, sex and Disease consequence. Segmented regression models were used to analyze the trend of tuberculosis changes. Also, data analysis software- Join Point Regression version 5.0.2 was used for data analysis.

**Results:**

The results of evaluating the trend of tuberculosis from 2010 to 2019 showed no change in the general trend of tuberculosis and an annual 0.84% (95% CI: ‐5.17 to 6.82) increase in incidence rate is observed in the trend. Also, the findings of join point regression analysis show that between 2010 and 2013, an annual 18.10% (95% CI: 8.78 to 34.89) increase in the incidence of tuberculosis, and between 2013 and 2019, annual −5.42% (95% CI: −10.04 to −2.22) decrease in the incidence of tuberculosis was observed. From 2010 to 2012, a 33.10% (95% CI: 15.77 to 48.06) annual increase in the incidence of tuberculosis in males and − 9.47% (95%CI: −14.02 to −6.33) annual decrease in the incidence of tuberculosis in females was observed.

**Conclusions:**

The results of this study showed that the incidence of tuberculosis had an upward trend from 2010 to 2013 and a downward trend from 2013 onwards.

## Introduction

Tuberculosis (TB) is the most common cause of death due to single-agent infectious diseases in the world and ranks tenth in terms of the global burden of disease [1]. In 2020, the global incidence of TB was 10 million (132 out of 100,000), which leads to an annual death of 1.3 million worldwide [2]. Additionally, the emergence of multidrug-resistant tuberculosis, caused by various gene mutations, is one of the growing public health concerns [3].

The World Health Organization's End TB Strategy recommends reducing TB incidences to <10 cases per 100,000 and less than one per 100,000 cases by 2035 and 2050, respectively, and eventually eradicating the disease [4]. In the era of globalization, there is no TB-free region, so without a global approach, eradication of this contagious disease is not possible in any region. >95% of cases and deaths from tuberculosis occur in low- and middle-income countries especially in African countries, so the burden of the disease is a principal public health issue [5]. However, it continues to be a persistent problem in high-income countries and among various vulnerable populations in every society [6,7].

The incidences of tuberculosis in the Eastern Mediterranean region and Iran were 114 and 16 cases per 100,000 people, respectively, which has a decreasing trend compared to previous years [[8], [9], [10]]. World Health Organization reports show various geographical distribution of tuberculosis in different parts of the world [11].

Epidemiological studies have indicated that several factors such as injecting drug use, smoking, which plays an important role in the recurrence of tuberculosis and increasing the death rate, consumption of >40 g of alcohol per day, which weakens the immune system and activates tuberculosis, malnutrition, BMI <18.5, and vitamin D deficiency can affect the increasing risk of developing TB [12]. Also suffering from diseases such as silicosis, diabetes, kidney failure, various cancers, gastric surgery, corticosteroids (>15 mg prednisolone daily for more than a month) or TNFα inhibitors, and celiac disease and patients who have undergone bone marrow or organ transplants are also prone to the disease [[13], [14], [15]].

Performing treatment short-course and BCG vaccination has been started in Iran since 1984 to control TB. In 1995, the DOTS strategy replaced the treatment short-course [16]. However, like many other countries, TB remains the major cause of health problems in Iran.

The present study aimed to evaluate the trend of TB disease incidence between 2010 and 2019 in the cities under the auspices of Ahvaz Jundishapur University of Medical Sciences. Disease trend analysis is one of the methods of epidemiological analysis used for monitoring, control, prediction, program review, policy analysis, and etiology of diseases. Reviewing the process of indicators and examining their changes allows health planners to evaluate the performance of the health system over different periods and to determine to what extent the implementation plans, along with the use of health and medical facilities and human and money resources will help us achieve our goals and what consequences they will have for solving our health problems. Also, determining changes in the trend of a disease incidence can be an effective tool for evaluating the efficiency and effectiveness of health control programs, measures taken, performance of healthcare staff, and decision-making for health planning [16,17].

## Materials and methods

### Study population

The population under this repeated cross-sectional study was census. In this study, we provided the incidence rates of TB cases from 21 March 2010 to 21 March 2019, following WHO guidelines derived from the reports published by the Deputy Health Minister of Khuzestan Province in the southwest of Iran, which hosts the national TB surveillance system. Due to the unavailability of information on HIV-positive cases, they were excluded from the study**.** The study area consisted of 21 cities of Khuzestan province with a population of over 3,519,713 million inhabitants (2022 population census).

Currently, there are 216 free TB diagnostic and treatment centers in this province. These centers quarterly report data from verified TB cases to the “Provincial Infectious Diseases Management Center, using electronic forms. In addition, annual reports on findings for TB control activities are provided for national use.

### Statistical analysis

In this study, we included an epidemiological indicator (coefficient of incidence per 100,000 inhabitants) and three variables (age, sex and Disease consequence).The incidence rate has been calculated according to the number of reported new cases each year and by defining the at-risk population as the total population covered for each year based on the data of the Provincial Infectious Diseases Management Center (Eq. 1).

(1) $$\mathrm{incidence rate of}\;\text{tuberculosis}=\frac{\mathrm{number of reported cases}}{\;\mathrm{total population}\;\mathrm{at}\;\mathrm{risk}}\;100000$$

In this study, the join point regression model (segmented regression model) was used to investigate the trend of tuberculosis from 2010 to 2019. The method of regression of connection points is used to analyze the trend of a disease over several years. This model can identify points where significant changes occur in the data process. This model finds breakpoints where the dependent variable has changed significantly at different times. The join point regression model is as follows:

$$E\left(y|x\right)=\beta_{0}+\beta_{1}x+\delta_{1}{\left(x-\tau_{1}\right)}^{+}+\ldots +\delta_{k}\left(x-\tau_{k}\right)$$

In this formula, x is the time variable, and y is the response variable. *β* and *δ* are regression parameters. β_0_ is the constant coefficient, and β_1_ is the slope coefficient. *k* is the unknown number of change points, *τ* is the unknown change points [18,19]. In this study, the incidence of tuberculosis was a dependent variable, and the year (time) variable was an independent variable.

For data analysis, Joinpoint Regression version 5.0.2 software, specially designed for joinpoint regression analysis, was used. The model used to find the number of connection points is the Weighted BIC (WBIC) model. If we want to have the best performance on average performs best across a wide range of situations, we use the weighted BIC method. Weighted BIC is the most flexible model in adapting to different situations.We used Standard Error to analyze Homoscedasticity with correlation structure “=First Order Autocorrelated with Correlation=0.05”.

Annual Percent Change (APC) is one way to characterize trends over time. With this approach, rates are assumed to change at a constant percentage of the rate of the previous year. Average Annual Percent Change (AAPC) is a summary measure of the trend over a pre-specified fixed interval. It allows us to use a single number to describe the average APCs over a period of multiple years. APC and AAPC quantities, the annual percent changes, and the average annual percent change, respectively, were calculated for the trend of tuberculosis in Khuzestan province. The APC indicates how much the incidence of tuberculosis has decreased or increased each year, and the AAPC shows the average annual change. If there is no trend change point, the value of these two quantities is equal. In this study, the incidence of tuberculosis was a dependent variable, and the year was an independent variable.

## Results

Table 1 shows that from the beginning of 2010 to the end of 2019, 5434 people were diagnosed with tuberculosis. In 2012, the least infected people and in 2019, the most infected people were identified. Also, during these ten years, the highest number of identified individuals were in the age group of 21–30 years, and the lowest were in the age group of below ten. During this period, 2639 had positive smear pulmonary tuberculosis, and 744 had negative smear pulmonary tuberculosis. In total, the highest and lowest incidence rates were 19.34 in 2014 and 12.91 in 2010 per 100,000 people. In males, the highest and lowest incidence were 23.63 in 2013 and 13.78 in 2010 per 100,000 people, respectively, and in females, the highest and lowest incidence were 16.29 in 2011 and 8.77 in 2019 per 100,000 people, respectively.

**Table 1:** Incidence Rate and frequency of tuberculosis based on demographic and clinical factors from 2010 to 2019.

Variable												
year		2010	2011	2012	2013	2014	2015	2016	2017	2018	2019	Total
age	0–10	5	3	9	3	5	3	9	3	6	11	57
	11–20	71	61	45	47	54	45	28	22	29	30	432
	21–30	131	174	168	157	133	138	146	134	107	75	1363
	31–40	115	139	146	143	133	151	137	134	112	105	1315
	41–50	66	64	87	80	66	83	75	82	85	65	752
	51–60	71	61	68	63	81	62	68	62	64	63	663
	61–70	33	38	43	40	56	41	44	51	43	39	428
	71–80	43	34	22	31	22	35	25	22	26	22	282
	80+	11	8	11	14	12	14	16	23	17	16	142
	Total	545	582	599	578	562	572	548	533	489	426	5434
gender	males	298	324	355	341	334	364	345	349	326	309	3345
	females	247	258	244	237	228	208	203	184	163	132	2104
Disease consequence	Pulmonary tuberculosis smear positive	262	298	298	259	269	268	270	256	257	202	2639
	Pulmonary tuberculosis smear negative	69	48	74	73	79	85	93	79	64	80	744
	Treatment failure	12	6	6	12	8	4	4	3	12	10	77
	Recurrence of the disease	27	0	22	29	24	20	25	26	17	17	207
	MDRTB	3	10	3	2	1	4	4	1	2	1	31
Incidence rate	males	13.78	14.82	22.57	23.61	22.63	21.93	21.87	22.92	21.33	20.15	
	females	12	12.36	16.29	16.93	15.95	12.94	13.36	12.43	10.85	8.77	
	Total	12.91	13.75	19.51	20.32	19.34	17.51	17.69	17.75	16.39	14.02	

**Fig. 1:**
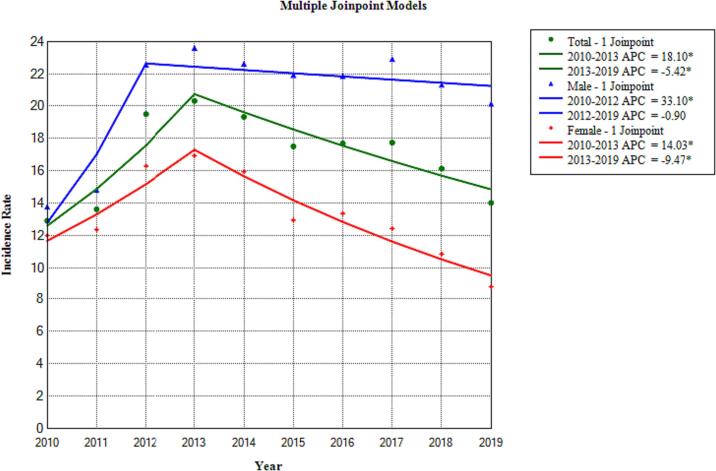
Annual Percent Changes (APC) of Tuberculosis Incidence in Khuzestan, Iran; 2010–2019.

Findings of joinpoint regression analysis to evaluate the trend of tuberculosis in all the covered people in the study period showed no identification of changes in the overall trends of tuberculosis and an annual 0.84% (95% CI: −5.17 to 6.82) increase in incidence rate is observed in the trend. Also, based on the findings of the joinpoint regression analysis in 2013, a change in the incidence of tuberculosis was observed. It divides the period under study into two parts. First, from 2010 to 2013, an annual 18.10% (95% CI: 8.78 to 34.89) increase in the incidence of tuberculosis is observed.Second, from 2013 to 2019, an annual −5.42 (95% CI: −10.04 to −2.22) decrease in the incidence of tuberculosis is observed (Table 2 and Fig. 1).

**Table 2:** APC and AAPC in tuberculosis patients in Khuzestan from 2010 to 2019.

	Trend 1		Trend 2		2010–2019
	Period	APC (95% CI)	Period	APC (95% CI)	AAPC (95% CI)
Total	2010–2013	18.10 (8.78 to 34.89) ^⁎^	2013–2019	−5.42 (−10.04 to −2.22) ^⁎^	1.85 (−0.56 to 4.40)
Male	2010–2012	33.10 (15.77–48.06) ^⁎^	2012–2019	−0.90 (−3.85 to 1.14)	5.81 (3.70 to 8.08) ^⁎^
Female	2010–2013	14.03 (5.30 to 28.21) ^⁎^	2013–2019	−9.47 (−14.02 to −6.33) ^⁎^	−2.23 (−4.63 to 0.0.02)

Abbreviations

95% CI: 95% Confidence Interval;

AAPC: Average Annual Percent Change;

APC: Annual Percent Change;

*The APC and AAPC are significantly different from zero (*p* < 0.05).

Findings of joinpoint regression analysis to evaluate the trend of tuberculosis in males in the study period showed no identification changes in the overall trends of tuberculosis and an annual 3.44% (95% CI: −2.78 to 10.02) increase in incidence rate is observed in the trend. Also, the findings of join point regression analysis show that in 2012, there was a change in the incidence of tuberculosis in males. It divides the study period under study into two parts. From 2010 to 2012, an annual 33.10% (95% CI: 15.77 to 48.06) increase in the incidence of tuberculosis and from 2012 to 2019, an annual −0.9% (95% CI: −3.85 to 1.14) decrease in the incidence of tuberculosis.

Findings of joinpoint regression analysis to evaluate the trend of tuberculosis in females in the study period showed no identification of changes in the overall trends of tuberculosis and an annual −2.74% (95% CI: −10.89 to 4.93) decrease in incidence rate is observed in the trend. Also, the findings of join point regression analysis show that in 2013, there was a change in the incidence of tuberculosis in females. It divides the study period into two parts. From 2010 to 2013, an annual 14.03% (95% CI: 5.30 to 28.21) increase in the incidence of tuberculosis and from 2013 to 2019, an annual −9.47% (95% CI: −14.02 to −6.33) decrease in the incidence of tuberculosis (Table 2 and Fig. 1).

## Discussion

In the present study, the trend of tuberculosis has changed over the past ten years. The overall incidence of tuberculosis in the cities under of Khuzestan Province has been increasing until 2013 and has been decreasing since then, which was generally consistent with previous studies in Iran [20,21]. The incidence of this disease in males and females during 2010–2012 and 2010–2013 had an increasing trend. Also, from 2013 to 2019, this rate had a decreasing trend in females and a steady trend in males. Our results from the higher incidence of tuberculosis in the male population showed that in all age groups, men are more likely to develop tuberculosis than women. Although a definitive explanation is not easy, men are more likely to be exposed to tuberculosis in society. However, regarding the risk of latent tuberculosis infection, a previous study indicated that women are at higher risk than men and 57.5% of cases of latent tuberculosis infection were among women [22]. Therefore, it is hypothesized that men have higher underlying risk factors for developing tuberculosis than women. Previous studies on the characteristics of active tuberculosis cases have reported that underlying conditions or diseases such as homelessness, HIV complex cases, and diabetes mellitus are more observed in the male population [5,23].

The present study was conducted using regression models and Joint Point software to investigate the incidence rate during different years. One of the main advantages of this method compared to other common methods in trend examination is the detection and determination of trend change points, thus providing separate processes for a while. Therefore, the examination of the tuberculosis trend for ten years in this study is influenced by various interventions and events during this period, such as DOTS treatment programs or the emergence of an AIDS epidemic, which can partially or generally justify the disease trend [21].

The global study reported an accelerating decreasing trend in developed countries with a high sociodemographic index. From 1990 to 2005 and 2005 to 2015, the average annual rate of changes in the age-standardized incidence and mortality rate decreased from −1.1% to −3.1% and − 1.1% to −7.2%, respectively [24].

In a study by Kazemnejad et al. on the tuberculosis incidence trend and mortality worldwide, the AAPC showed a declining trend for these two rates from 2001 to 2010, both worldwide and in all WHO regions. They also found a significant increase in the diagnosis of tuberculosis patients worldwide for five WHO regions [20].

In a study reviewed by Salman Khazaei et al., it was found that the incidence and mortality of tuberculosis in people with HIV had been increasing from 1990 to 2014. In this regard, the largest increase was from 1995 to 2001 in the incidence rate and from 1996 to 2005 in the mortality rate. Also, no significant trend in case detection was observed throughout the study period [21]. Ibrahim al-Orayini and colleagues assessed the tuberculosis incidence trend in Saudi Arabia from 1991 to 2010. The trend of tuberculosis was increasing in the first ten years of the study and then decreased slightly. The higher the age, the more incidence, but it showed a decreasing trend for people over 45 years [25]. According to the findings of a study conducted in Brazil by Brito et al., the incidence of tuberculosis was stable between 2001 and 2004 and decreased from 2004 to 2016 [26].

A decreasing trend in tuberculosis cases registered in Fiji has been reported by Tamami et al. Along with other countries such as Fiji, this decrease was partly due to various interventions such as BCG vaccination, initiation of primary health care (PHC), and lavatory and radiography facilities [27]. In a study of tuberculosis trends by Rahimi et al., in West Azerbaijan province, it was reported that the incidence rate decreased before 2003 and then slightly increased with the highest slope in 2008 [28].

There is a growing trend of TB and HIV worldwide, which strongly links TB and HIV. The most common opportunistic infection and cause of death in people infected with HIV worldwide is tuberculosis. HIV infection can affect the incidence and mortality of tuberculosis in different ways, including the destruction of tuberculosis treatment, the increased risk of TB recurrence and side effects or mortality due to treatment, and the increased risk of TB transmission [29]. In a 10-year study, it was reported that in many African and Asian developing countries, the number of tuberculosis patients has increased 2 to 3 times due to the high prevalence of HIV / AIDS [30].

There are several limitations to our study. First, incomplete data from individuals and variables on demographic and behavioral factors, and not collecting socio-economic factors. However, in new registry systems, these factors are included, which makes explaining the trends difficult. Second, studies of TB trend at the country level is too low, which makes it difficult to compare the results. The segmented regression is inadequate to regress data including outliers, so it is necessary to extend this model to overcome the outlier problems**.**

## Conclusions

In summary, the incidence trend of tuberculosis in Khuzestan has been increasing until 2013 and has been decreasing since then, which was generally consistent with previous studies in Iran.

## Ethics approval and consent to participate

Because the data collection method was observation and there were no human participants in the current study, according to regulations obtaining informed consent is deemed unnecessary; The Ethics Committee of Ahvaz Jundishapur University of Medical Sciences (AJUMS.REC.1399.445) confirmed the morality and ethics of the study.

## Consent for publication

Not applicable.

## Authors' contributions

Conceptualization, NA; Methodology, NA, BC and SA. Software, NA, and ME; Formal.

Analysis, NA and ME.; Investigation, BC, NA, and ME.; Resources, NA.; Data Curation, SA, ME, and NA.; Writing – Original Draft Preparation, NA.; Writing – Review & Editing, NA, SA, BC, ME and SA.; Visualization, NA.; Supervision, SA and NA.; Project Administration, NA.

## Declaration of Competing Interest

The authors declare that they have no competing interests.

## Data Availability

The datasets used and/or analyzed during the current study are available from the corresponding author upon reasonable request.
